# What Have We Learned about the Prevention of NMSC from Albino Patients from Malawi? Secondary Prevention Maintained over Time

**DOI:** 10.3390/cancers16081522

**Published:** 2024-04-16

**Authors:** Alejandra Tomás-Velázquez, Ester Moreno-Artero, Javier Romero, Pilar Escalonilla, Isabel Medina, Gisela Hebe Petiti, Pedro Redondo

**Affiliations:** 1 Dermatology Department, Clínica Universidad de Navarra, 28027 Madrid, Spain; predondo@unav.es; 2 Dermatology Department, Hospital Universitario Galdakao, 48960 Galdakao, Spain; ester.morenoartero@osakidetza.eus; 3 Romero y Medina Dermatology Clinic, 29640 Fuengirola, Spain; dr.romero@romeroymedina.com (J.R.); dra.medina@romeroymedina.com (I.M.); 4 Dermatology Department, Complejo Asistencial de Ávila, 05071 Ávila, Spain; pescalonilla@saludcastillayleon.es; 5 Dermatology Department, Consorci Sanitari Integral, Hospital Dos de Mayo, 08025 Barcelona, Spain; giselahebe.petiti@sanitatintegral.org

**Keywords:** albinism, skin cancer, squamous cell carcinoma, Africa, Malawi, prevention, diagnosis, treatment, surgery

## Abstract

**Simple Summary:**

Albino patients in rural Africa have a very high risk of skin cancer. We have conducted several cooperative campaigns in recent years focusing on the care of this population in a rural area of Malawi and have reached some important conclusions. Primary prevention is fundamental and, in addition to education, providing adequate clothing offers more sustainable and long-lasting photoprotection and is more effective than offering sunscreen sporadically. However, at present, secondary prevention focused on frequent follow-up and early diagnosis of treatable lesions is what really makes the difference in many patients affected by squamous cell carcinoma, who otherwise will eventually die. To achieve this, it is essential to have funding and local collaboration with a comprehensive organization prior to visits by using teledermatology and frequent campaigns of qualified health staff who, in addition, help in the training of local personnel.

**Abstract:**

Background: We have conducted cooperative campaigns focusing on albino patients in a rural area of Malawi. What have we learned? Methods: Three surgical campaigns were performed in Nkhotakota district (2019–2023). Albino clinical and tumor characteristics were collected. Results: Between 22 and 75 albinos were evaluated in each campaign (mean age < 28 years old). Most patients did not use sunscreen in a way that provided optimal photoprotection. Regarding tumors, the proportion of basal and squamous cell carcinomas ranged from 1:1 to almost 2:1. Of 156 albino patients, 34 attended more than once. However, of the 19 patients with 30 tumors operated on in 2021, only seven were assessed the following year (12 were lost to follow-up). At least 14 albinos with locally advanced tumors were evaluated. Conclusions: Distributing photoprotective clothing could be more efficient or perhaps an earlier measure of sunscreen in rural Africa as it does not require permanent repositioning. Very-high-risk patients (previous interventions with positive margins or high-risk tumors, intense actinic damage, and new tumors constantly appearing, especially those presenting SCCs) require close follow-up and treatment and represent our main target. Secondary prevention with Malawian collaboration and the use of teledermatology is essential for patient tracking, as they are able to offer curative treatments.

## 1. Introduction

Albinism is a heterogeneous genetic disorder caused by mutations in different genes, which leads to a reduction or total absence of melanin in the skin, hair, and eyes. In the eyes, the lack of melanin in the iris and retina during embryogenesis may lead to multiple ocular complications such as macular dysplasia, hypoplasia of the fovea, or chiasmatic abnormalities of the optic nerve, among others, which normally lead to severe abnormalities in visual acuity, abnormal stereoscopic sight, refractive errors, severe photophobia or nystagmus [1,2,3,4,5]. In the skin, it leads to greater susceptibility to solar radiation and an increased risk of skin cancer. The most frequent form is epidermoid or squamous cell carcinoma (SCC), followed by basal cell carcinoma (BCC). In contrast, cutaneous melanoma is rare [6,7,8]. SCC is the main cause of death in albino patients in Africa [9] and, indeed, the risk of SCC in black albinos is one thousand times greater than in the general population, and head and neck are the areas most frequently affected [10]. Furthermore, in albino patients, particularly in areas exposed to the sun, the cancer has a more aggressive course and a higher recurrence rate than in people with normal pigmentation, whether they are white or black [11,12,13,14]. It is estimated that 100% of albino patients in African countries develop precancerous lesions of the skin before the age of 20 and have a mean life expectancy of 30 years. This means that, by the third decade of their life, many black albinos in Africa will have developed a potentially lethal SCC [13,14].

The estimated overall prevalence in the population is 1/17,000 inhabitants [1,2,3,4,5]. In sub-Saharan Africa, according to the World Health Organization, this prevalence is higher, reaching from 1/5000 to 1/15,000. OCA type 2, caused by *OCA2* variants, is the most frequent form of albinism in African countries. According to the 2018 Malawi Population and Housing, of the total population, about 0.8% (134,636) were people with oculocutaneous albinism (OCA), although reports from NGOs and related associations state that there are no more than 7000 people with OCA in Malawi; the lack of resources, infrastructure, education, and health care makes them a community that is extremely susceptible to developing skin cancer.

Several studies have shown the importance of photoprotective measures to reduce the incidence of nonmelanoma skin cancer (NMSC), both in normal immunocompetent populations and high-risk patients, whether they are immunosuppressed, such as patients having undergone solid organ transplantation, or those with genodermatosis, such as albinos or those suffering from xeroderma pigmentosum. Primary prevention basically consists of educating patients to avoid direct exposure to sunlight, the application of sunscreen, and the use of photoprotective clothing.

However, the experience gained from three surgical campaigns aimed at treating NMSC in albino patients in Malawi has opened our eyes to the failure of the prevention of NMSC in a large number of patients. In Malawi, most of our patients live in a rural environment, are extremely poor, and have only their most basic needs satisfied. Many of them are peasants and make their living by working the land. Most have access to sunscreen only as a result of cooperation campaigns. Many of these campaigns periodically distribute sunscreens to albino patients, but hardly in sufficient quantity to guarantee their daily use throughout the year.

Evidence shows that, while the same social situation continues, secondary prevention is more important than primary prevention, at least in a group of patients: early diagnosis and the possibility of prompt treatment. In the course of these campaigns, we detected, in our working area, at least 14 young patients with locally advanced or metastatic NMSC. Although it is true that the period between the second and third campaigns was marked by the COVID-19 pandemic, which led to a significant reduction in the number of international cooperation campaigns, the incidence is very high and shows how preventive measures have failed in those patients.

## 2. Materials and Methods

Between 2019 and 2023, three surgical campaigns were run under the umbrella of the Dermalawi cooperation project (www.dermalawi.com accessed on 16 April 2024), which is organized in the Benga area (the Nkhotakota district) with the cooperation of Spanish dermatologists (Table 1). In the first campaign, in August of 2019, which was initially focused on surgery for skin tumors, it was confirmed that malignant tumors were mostly restricted to albino patients. For this reason, the campaign was redirected in situ toward this group of patients, and the two following campaigns (in August 2021 and January 2023) were aimed expressly at the follow-up and treatment of NMSC in albino patients. Thus, a triage was performed among all the patients who could attend. Data about albino clinical characteristics and tumors (including treatment and the pathological results in case of the surgeries) have been collected.

## 3. Results

The number of albino patients evaluated in each campaign was variable and ranged from 22 to 75 (Table 1). There were no significant differences between sexes and the mean age was under 28 years (Table 2). We observed that most patients did not have enough sunscreen nor were they in the habit of applying it with the frequency and amount of product necessary to achieve adequate photoprotection.

The proportion between BCC and SCC ranged from 1:1 to almost 2:1, far from the greater proportion in immunocompetent patients (5:1) (Table 2). Some different lesions (neither BCC nor SCC), most of them benign, were excised or biopsied during the campaigns: four in 2019, five in 2021, and five in 2023. Pathological reports confirmed ulceration and/or solar elastosis (5), melanocytic nevi (4), scar (1), actinic keratoses (1), solar lentigines (1), Kaposi sarcoma (1) and dilated pore of Winner (1).

The analysis of the first surgical campaign showed a selection bias with an overestimation of actinic keratoses and tumors, given that, as the campaign was designed exclusively for surgery, albino patients with greater actinic damage and tumors were treated, while those without self-detected lesions did not come for assessment. The following campaigns were aimed at all the albino patients in the area, and we included younger patients and children many of who did not have skin lesions. However, we did observe young patients (<18) presenting intensive sun damage, actinic keratosis, and even SCCs.

Of a total of 156 albino patients, 34 attended more than once, which shows at least partial adherence to the NMSC prevention and treatment program. However, of the 19 patients with 30 tumors operated on in August 2021, only seven were assessed the following year, and thus, 12 of them were lost to follow-up (Table 1). Furthermore, there is a percentage of resected tumors with positive histologic margins (*n* = 10, 15%) which will require closer follow-up, especially the cases of SCC (Table 3). Moreover, the number of albinos with locally advanced tumors who were not candidates for surgery may be underestimated; in the campaigns, 14 patients were evaluated in person, but there are at least another three patients who were assessed by teledermatology and whom we have never actually seen.

## 4. Discussion

For years, melanoma has been the paradigm of dermatologic urgency due to the need for diagnosis and early surgical treatment in skin oncology. However, dermatologists who dedicate themselves to dermato-oncology know that an SCC often has the ability for local destruction and a greater aggressiveness and, therefore, requires greater priority as a surgical urgency, especially those that appear on the cephalic area.

The main reasons for the NMSC prevention failure in Malawian albinos are that Malawi is a developing country with a health care system that could be improved, the education in primary prevention is probably deficient, patients have poor awareness of the disease, and the means to carry out specific follow-up for an early diagnosis do not exist, nor the possibility to treat patients with priority. To this, we add the social stigma attached to albinism in Africa. This stigma, as their condition is clearly visible, leads to a situation where albinos are easily isolated in rural environments and avoid socializing in towns or larger cities [15,16,17]. This lack of social interaction favors, even more, the delay in diagnosis as those people may tend to consult late or not at all, even when the tumors appear in visible and photoexposed areas such as the cephalic area. Thus, it is easy to understand what a lethal cocktail it can be to develop an SCC, especially in the cephalic area, in Malawi. 

Comparing black African albino patients with European albino patients, it is worth mentioning that, when analyzing the scientific literature, the vast majority of cases of skin cancer in albinos are circumscribed to African countries [2,3,4,5,6,7,10,11], probably because of the intensity of UV radiation in these latitudes, less accessibility to sun protection methods and therapeutic education, and as a result of the outdoor farming activities and lifestyle in African rural areas.

Current cooperation campaigns, with the collaboration of pharmaceutical companies, focus on the distribution of sunscreen. Although this helps to raise awareness of the disease and the risks that come with it, our feeling is that, in practical terms, to supply an albino patient with a couple of containers of sunscreen that in no way covers their real medium and long-term photoprotection needs, it is not enough. The efforts to manufacture and produce sunscreen locally are highly praiseworthy, provided that they are accompanied by free and permanent distribution to the groups at risk. If such requirements are not met, however, we are back to square one.

Therefore, among all the primary prevention measures, the most important is to educate patients about the harmful effects of exposure to sunlight, without forgetting the context of the daily social reality experienced by albino patients. Secondly, between the continuous application of sunscreen and the use of photoprotective clothing (wide-brimmed hats or caps with lateral flaps), it would seem a priori that this latter measure would be more feasible and effective in rural Africa, as the clothing can be distributed periodically in cooperation campaigns and is not fungible and, thus, does not require permanent reposition. 

In addition to the above, it is mandatory to perform secondary prevention, combining resources and efforts to allow early diagnosis and treatment. Undoubtedly, the socioeconomic development of the Malawian population and the training of dermatological physicians and surgeons will make a real difference, thus, our campaigns must include helping to train local health workers. Meanwhile, in the technological decade in which we live, especially in the development of teledermatology, it can be feasible to make early diagnoses remotely. But, for this, it is necessary to have personnel, medical or otherwise, available locally who evaluate patients periodically in reference centers or move expressly to the rural areas, allowing an adequate follow-up. Our experience is that this situation is possible if economic funding exists to maintain it. 

Many cooperation campaigns focus on providing aid at one particular point in time, which is not efficient if the local preparatory and coordinating infrastructure is not in place. For example, our first surgical campaign served to show that the priority was albino patients, but that it was very important to coordinate the localization, movement, and triage of the patients very well, according to their severity and surgical need in order to be treated within the established time period. Unfortunately, also in the second campaign, some patients attended late or required unforeseen surgery, which needed unavailable resources and thus, could not be performed. There is always a percentage of unforeseen events in this type of campaign that must be considered, but the fewer there are, the fewer patients will probably not receive treatment due to lack of time or resources (such as an anesthesiologist or specific material). 

Furthermore, the project requires the professional altruistic involvement of a group of dermatologists who follow patients remotely and have the possibility of being able to travel themselves or coordinate the travel of other colleagues to carry out the surgical campaigns. Our experience of these 3 years is that there is a large group of dermatologists in Spain who are willing to collaborate or become personally involved in this challenge. Ideally, several campaigns, strategically separated in time, should be planned each year, and, whenever possible, visits and surgeries should be organized prior to the trip using the reception of images. Collaboration with other cooperation groups will allow the referral of urgent patients whose surgery should not be delayed.

Patients who have received a solid organ transplant have a greater predisposition to developing NMSC, especially SCC. In our countries, all of them are subject to secondary prevention: periodic protocolized follow-up for diagnosis and early treatment. Although we encourage primary prevention, avoiding exposure to sunlight and following photoprotective measures, secondary prevention is essential.

This is the same that should be implemented with albino patients; they must be assessed every 4–6 months, and the way to carry this out at present in rural Malawi is by teledermatology. Most tumors are located on the cephalic pole (head and neck) and thus, taking a series of photographs of this location would be fundamental. Ideally, for the examination of the full body surface, a clinical officer—the name given to highly qualified health care personnel in Malawi, few in number—should be available, but if this is not possible, an unqualified health care collaborator could be trained to take these images, at least centered on the cephalic pole. Therefore, a trained and contracted person fully dedicated to the project who periodically travels through the villages with a mobile telephone and the ability to take quality images and who can communicate with dermatologists remotely, is needed.

As in immunosuppressed patients, SCC in albinos follows a more aggressive course and has a higher recurrence rate than in patients with normal pigmentation [11,12,13,14]. Cyclobutane pyrimidine dimers (CPDs) formation is responsible for most skin tumors. UV-B radiation (290–320 nm), responsible for sunburn, directly damages DNA through the formation of CPDs. In addition, UV-A is able to induce the production of superoxide and nitric oxide, which excite melanin and transform it into its high-energy derivatives. This high-energy melanin transfers its energy to DNA, creating mutagenic CPDs within hours of exposure to UV-A radiation. It seems that pheomelanin, the predominant melanin in fair-skinned people, such as albino patients, is a more potent generator of dark CPDs than eumelanin [18].

Therefore, in the campaigns, apart from new patients, it is fundamental to review patients who are already known or who have undergone treatment in order to rule out local relapse and/or the appearance of other neoplasia.

**Figure 1:**
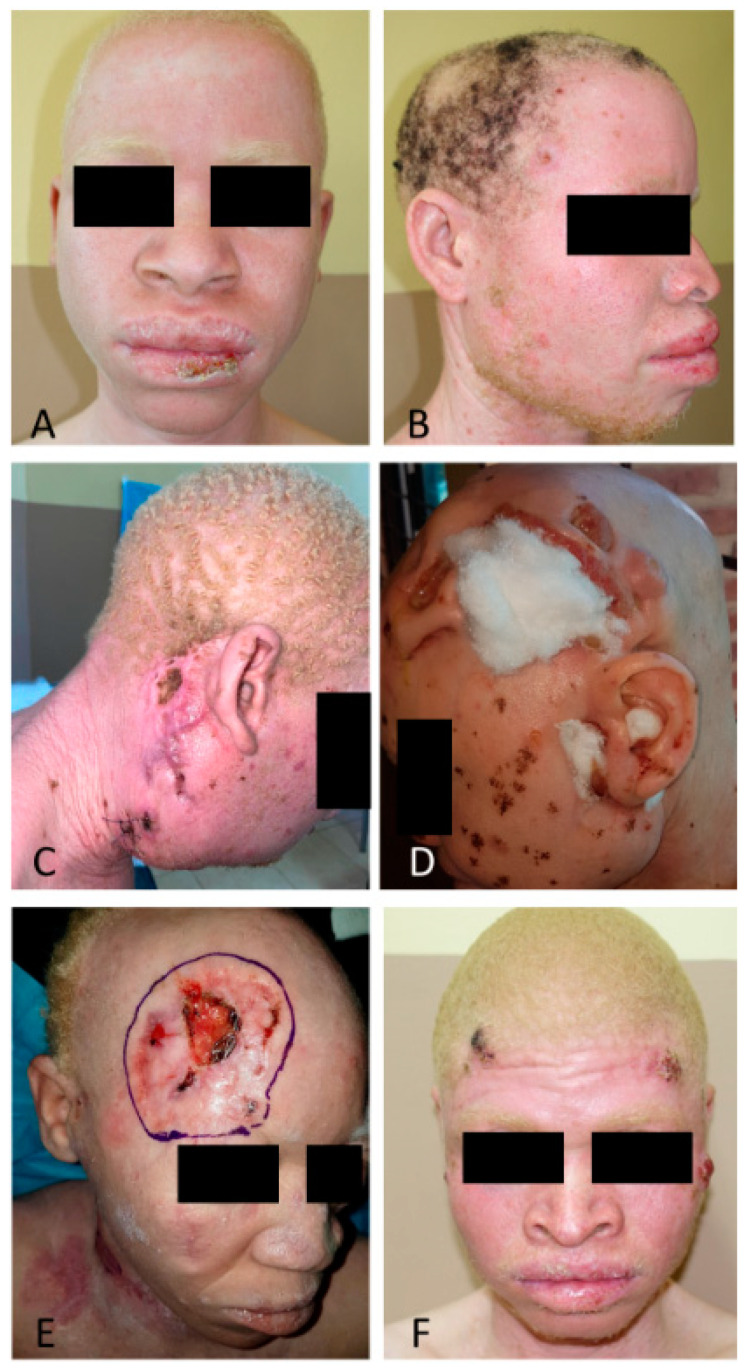
Albino patients of different risks and phenotypes, consulting for skin tumors of different stages. Patients depicted in (**A**,**B**) are young patients, presenting with tumors that can be cured with surgical treatment and, if they undergo primary prevention and adhere to follow-up campaigns, they can have a good outcome. (**C**,**D**) illustrate patients assessed with locally advanced or metastatic tumors, not susceptible to curative treatment in Malawi; and not assessed for surgical treatment at previous times. (**E**,**F**) depicted high-risk patients who were able to receive surgical treatment for high-risk tumors. These patients require close follow-up for early diagnosis and treatment, otherwise they will have a poor outcome.

On the basis of our data, we can divide albino patients with NMSC into three groups (Figure 1). The first is made up of patients with incipient tumors that can be successfully resected. These patients are aware of the importance of attending follow-up and can be controlled in surgical campaigns. The second group is made up of high-risk patients with recurrent tumors who are known and ask for treatment but require closer follow-up because, in the present circumstances; they may progress if there are delays or loss of follow-up. The third group of patients consists of those at very high risk, who are probably not aware of the severity of their condition, who do not attend consultations, or when they do, leave it very late. From these last two groups come the patients with locally advanced tumors in whom, very frequently, no surgical action with curative intent is possible. Patients in the third group represent a priority and should receive the bulk of our efforts. There are patients who need to be sought out expressly in their environment so that they can move to the second group. Finally, all patients should receive regular follow-up in order to increase the degree of adherence to the campaigns progressively.

In the literature, there are scarce reports about the management of albino people in Africa. Recently, Wright et al. have published relevant information about the epidemiology and prevalence of skin cancer in Southern African countries and skin cancer prevention campaigns in those countries, especially for people with fair skin, or oculocutaneous albinism, and HIV-AIDS, who are at the greatest risk. They argue about the possible impacts of climate change on skin cancer in Southern Africa [19].

With regard to the limitations of our work, we highlight the impossibility of following up some patients who live far away or whom we have no way of contacting. These difficulties also mean that some patients who need it and would really benefit from our intervention never come to be evaluated as well as that some, due to lack of awareness, do not make an effort to come until the tumors are very advanced. In this regard, we are unaware of the evolution of some of the albinos who have undergone surgery or other treatments. In addition, we do not yet have feedback on the long-term effect of these interventions on NMSC prevention, which undoubtedly depends to a large extent on the involvement and training of the Malawian population and on education, especially in the youngest patients with less accumulated actinic damage, still in time to avoid much of it. While we have English to Chichewa translators for all campaigns, the language barrier and educative and cultural differences can limit the adherence to primary and secondary prevention measures, and can make it difficult for patients to understand their illness.

## 5. Conclusions

In conclusion, as regards primary prevention interventions, we believe that the most important is educating patients about the harmful effects of exposure to sunlight and the life-threatening risk that comes with it, while not forgetting the use of photoprotective clothing. This measure, as it is not a perishable intervention, should perhaps come before the use of sunscreen, as sunscreen supply should be constant and the application daily and regular, which is very difficult in the present circumstances. As far as secondary prevention is concerned, campaigns are necessary to search for and monitor albino patients, especially those at high risk, for the diagnosis and early treatment of SCC before such lesions become locally advanced or metastatic.

## Figures and Tables

**Table 1:** The number of albino patients assessed during the campaigns.

Adherence to Medical Follow-Up	2019	2021	2023	TOTAL
Number of albino visits	22	75	59	156
Number of albino patients already seen in previous campaigns	Not applicable	10	24 in total: 19 in 2021, 5 in 2019	34
Number of new albino patients registered	22	65	35	122

**Table 2:** The number and characteristics of albino patients seen during the campaigns, the number of surgeries performed per tumor, and the number of patients with locally advanced tumors.

Surgical Cooperation Campaigns for Albinos			2019	2021	2023
Number of albino patients seen			22	75	59
Gender Male: Female (%)			8 (36.4):14 (63.6)	40 (53.3):35 (46.6)	30 (50.8):29 (49.15)
Age (mean)			27.45 (11–50)	22.64 (2–61)	27.1 (3–62)
Solar lentigines *n* (%)			12 (54.5)	37 (49.3)	18 (30.5)
Actinic keratoses *n* (%)			20 (90.1)	33 (44)	43 (72.8)
Skin cancer	Number of tumors evaluated		14	32	35
	Number of tumors operated	SCC	6	11	10
		BCC	6	17	17
	Locally advanced tumors ^1^		2	4	8

^1^ At least three more patients with locally advanced tumors have been evaluated by teledermatology in recent years.

**Table 3:** Number of SCC and BCC per campaign and assessment of surgical margins.

Surgically Treated Tumors	2019		2021		2022		
**Margins**	**−**	**+**	**−**	**+**	**−**	**+**	**Unknown**
**SCC**	5	1	8	3	6	**−**	4
**BCC**	6	**−**	13	4	13	2	2

## Data Availability

The data supporting this study’s findings are available from the corresponding author upon reasonable request.
